# A Glimepiride-Metformin Multidrug Crystal: Synthesis, Crystal Structure Analysis, and Physicochemical Properties

**DOI:** 10.3390/molecules24203786

**Published:** 2019-10-21

**Authors:** Xufei Bian, Lan Jiang, Zongjie Gan, Xiaoshu Guan, Li Zhang, Linhong Cai, Xiangnan Hu

**Affiliations:** 1 Department of Medicinal Chemistry, School of Pharmacy, Chongqing Medical University, Chongqing 400016, China; bianxufei95@163.com (X.B.); 13320227532@163.com (Z.G.); 17082343045@163.com (X.G.); zhangli3357@163.com (L.Z.); cailinhong123@163.com (L.C.); 2 College of Environment and Resources, Chongqing Technology and Business University, Chongqing 400016, China; jianglanlucky@163.com

**Keywords:** Glimepiride, metformin, dissolution, hygroscopicity

## Abstract

A multidrug crystal based on drug combinations was synthesized by the solvent evaporation method. This multicomponent crystal consisted of antidiabetic drugs Glimepiride (Gli) and Metformin (Met), which was performed by single crystal X-ray structure analysis. The results showed an enhancement of the pharmaceutical properties such as lower hygroscopicity and greater accelerated stability than the parent drug Met, and a higher solubility and dissolution rate than Gli.

## 1. Introduction

In the field of pharmaceutical chemistry, the method of designing multi-target drugs [1] is being taken more and more seriously than developing multiple drugs that can bind to different target points in treating a particular disease. Compared to the laborious synthetic method of designing a new active pharmaceutical ingredient (API), co-crystallization technology is a chemical approach to bring together two or even more different molecules into a crystalline lattice using non-covalent interactions [2,3]. The multidrug crystals can not only alter the physicochemical properties such as dissolution rate, hygroscopicity, and stability of the involved APIs, but also offer improved pharmacological benefits [4,5,6,7,8].

Recently, there has been an increasing number of applications to combine oral drugs. A common combination of the type 2 diabetes mellitus (T2DM) drugs Metformin (Met) and Glimepiride (Gli) was founded after the screening of marketed combination drug formulations. Met, a biguanidine hypoglycemic drug, is the first choice for T2DM [9,10]. Additionally, Gli is the first-line drug for the long-term treatment of T2DM [11]. These two drugs are effective in the treatment of T2DM as both single and combined therapy [12].

However, both Met and Gli have some disadvantages of physicochemical properties. Met is commonly available as hydrochloride salt to address the high hygroscopicity in the base form [13,14]. Additionally, Gli has poor dissolution, which ranks as a class II molecule according to the Biopharmaceutical Classification System (BCS) [15]. Many methods have been adopted to tackle these problems during the manufacturing process, which is expensive and may increase the cost of the drug [16,17]. The synthesis of a multidrug crystal, a highly advanced technique, can rearrange the molecules to form a new crystal structure. This technology can be used to improve the physicochemical properties of parent drugs [18,19].

In this article, we designed the synthesis of a multicomponent crystal based on drug combinations of Gli and Met, which was performed by single crystal X-ray structure analysis. Additionally, the issue of the poor dissolution of Gli and the high hygroscopicity in Met was tackled by the formation of a multicomponent crystal. Last but not least, the preparation process of the Gli-Met is simple, and the reagent is cheap and easy to obtain, which can be used in industrial production.

## 2. Results and Discussion

### 2.1. The Characterization of Crystal Structure

**Figure 1:**
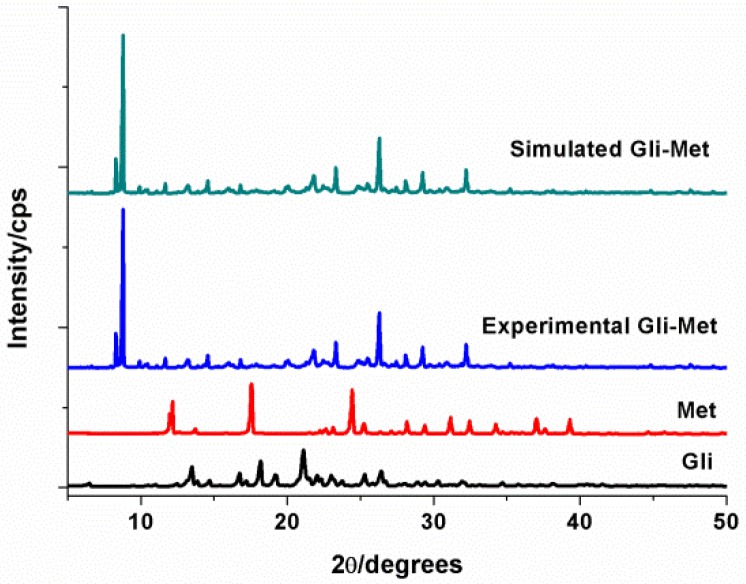
X-ray powder diffractograms of Gli-Met, Met and Gli.

With the simple and fast method of solvent evaporation, Gli-Met was successfully prepared for three days, which yielded up to 84.2%. At the first stage, powder X-ray diffraction (PXRD) and thermal analysis were used for the preliminary detection of the difference among Gli-Met and two raw materials. The diffractograms of Gli-Met, Met and Gli were characterized by PXRD. Obviously, when compared with the patterns of Met (13.2, 18.1, 24.9, 32.0, 34.7, 37.6) and Gli (14.2, 16.8, 18.2, 19.1, 22.4), those of Gli-Met exhibited differences that were characterized as 2θ at 9.4, 9.5, 11.2, 22.3, 23.6, 20.6 and 26.8 (Figure 1). The PXRD spectra of the Gli-Met demonstrated the disappearance of the characteristic peaks of both Met and Gli and the appearance of novel characteristic peaks. Moreover, the experimental PXRD patterns of Gli-Met well matched the simulated patterns generated from the corresponding single crystal, indicating that the prepared powders were of pure crystalline phases.

**Figure 2:**
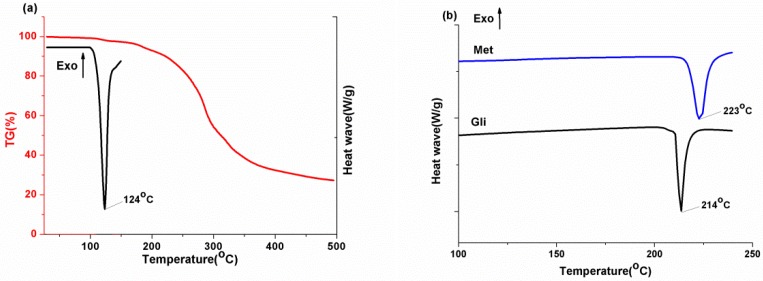
(**a**) Differential scanning calorimetry (DSC) and thermogravimetric analysis (TGA) thermograms of Gli-Met; (**b**) DSC thermograms of Met and Gli.

Thermogravimetric analysis (TGA) and differential scanning calorimetry (DSC) were employed to investigate the thermal properties of Gli-Met, Met, and Gli samples. Thermal analysis was a reliable method of characterization of APIs in terms of the structural and stability investigations. As shown in Figure 2, the TGA thermogram of Gli-Met shows a slight but continuous weight loss of 2.8 % before its melting point, indicating slow elimination of the crystal lattice water molecules. Furthermore, there were three exothermic peaks accompanied by chemical decomposition at 124, 214, and 223 °C, which were observed in the DSC thermograms for Gli-Met, Met, and Gli samples, respectively. These points corresponded to the melting point of the three materials.

**Figure 3:**
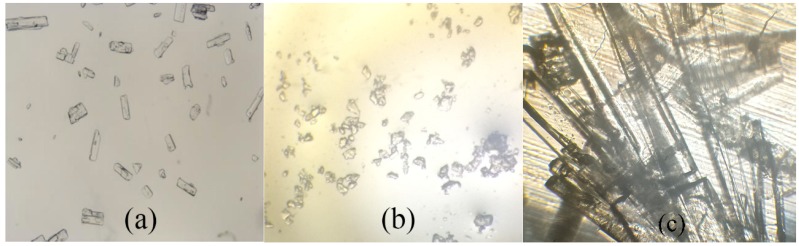
Micrographs of (**a**) Met (40×), (**b**) Gli (40×), (**c**) Gli-Met (100×) obtained by polarized optical microscopy (POM) at room temperature.

For further clarification, the micrographs of Met, Gli, and Gli-Met were characterized by polarized optical microscopy (POM). This technique allows for the easy identification of the presence of crystals. The three kinds of crystals exhibited different forms which are flake-shaped (Met), small block-shaped (Gli), and prism-shaped (Gli-Met). The images obtained are presented in Figure 3.

**Figure 4:**
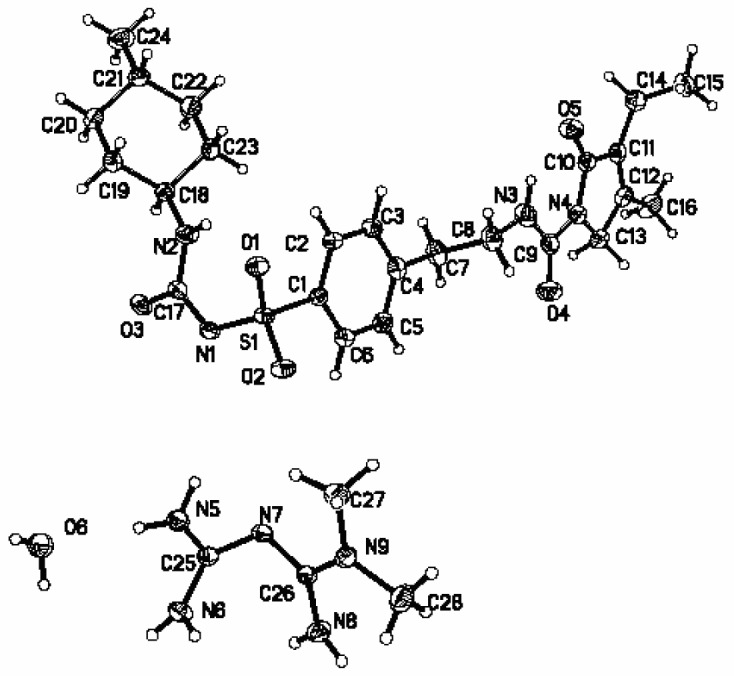
Thermal ellipsoid figure for the Met ion, Gli ion, and H_2_O molecules drawn at 50% probability level.

The single crystal of Gli-Met was analyzed by single crystal X-ray diffraction (SCXRD). The corresponding crystallographic data and refinement details are summarized in Table 1. The crystal structure of Gli-Met was in the monoclinic space group P2_1_/c: a = 30.9830 Å, b = 8.91714 Å, c = 11.8813 Å, α = 90°, β = 98.8439°, γ = 90°, T = 293 K, and the final R1 was 0.0470 (I > 2σ(I)) and wR2 was 0.1368 (all data). There was one molecule of Gli, one molecule of Met, and one molecule of crystal water (H_2_O) in the asymmetric unit in Figure 4.

**Figure 5:**
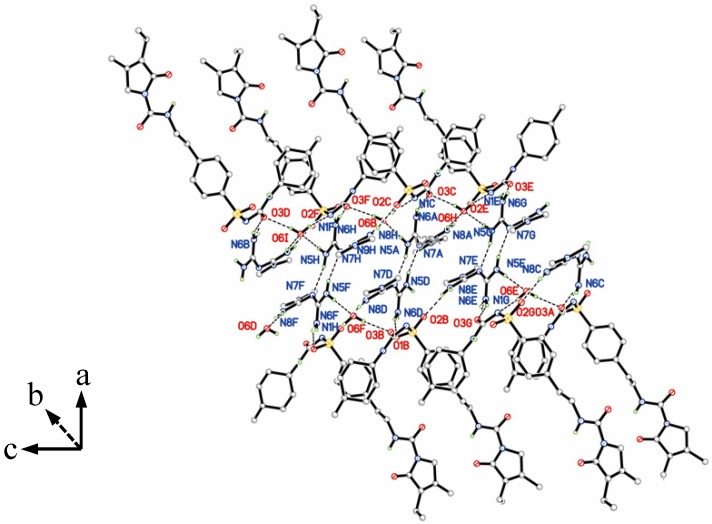
Two dimensional layered structure connected by hydrogen bonds of Gli-Met.

Gli-Met is characterized by complex 2D hydrogen-bonded layers parallel to (1 0 0), as illustrated in Figure 5 and Table 2. In the central section, two Met ions form a centrosymmetric homodimeric motif through N(5)-H(5B)…N(7) hydrogen bonds. Each Met ion interacts with two water molecules and two Gli ions via relatively strong hydrogen bonds. The interactions between Met and the water molecules are established by hydrogen bonds N(5)-H(5A)…O(6) and N(8)-H(8C)…O(6). The N(6)-H(6B)…O(3)_carbonyl_ and N(8)-H(8D)…O(2)_sulfonyl_ hydrogen bonds are responsible for the bonding between the Met and Gli ions. Additional hydrogen bonds between Gli and water, i.e. O(6)-H(6C)…N(1) and O(6)-H(6D)…O(3), strengthen the bonding of Met to the Gli molecules further. The interaction between every two molecules of Gli in the outwards was formed by the N(2)-H(2A)...O(1)_sulfonyl_ and N(3)-H(3A)...O(5)_carbonyl_ hydrogen bond.

From the prospective of hydrogen bonding analysis, all donor and acceptor moieties of hydrogen bonds in Met actually formed hydrogen bonds with the Met, Gli, and H_2_O molecules. This means that Met has the competence to become a first-line choice of the selection of the co-former during the preparation of the multidrug crystals. On the basis of these intermolecular interactions, the Met and H_2_O molecules were sandwiched by the Gli molecules and formed an infinite hydrogen bond chain along the b axis.

**Figure 6:**
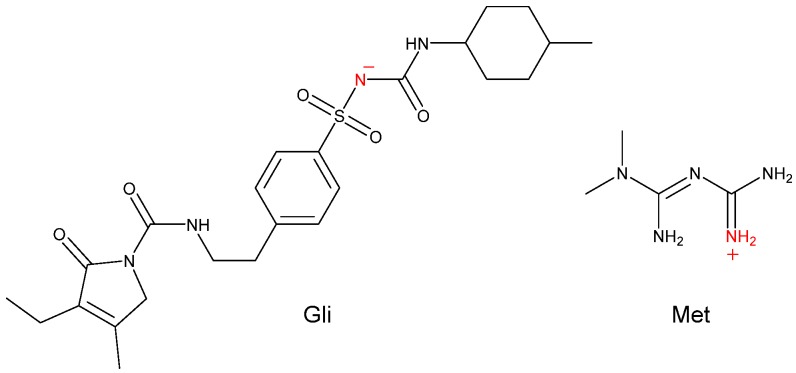
Chemical structures of the Gli and Met with basic and acidic sites highlighted, respectively.

Interestingly, the molecules of Gli and Met were connected not only by hydrogen bonds, but also by the ionic bond as shown in Figure 6. More specifically, the N-H hydrogen atom at the nitrogen labelled N1 between the sulfonyl group and carbamide group of Gli moved to the imine group of Met. Thus, the stability of the intermolecular structure was maintained by hydrogen bonds, ionic bonds, and van der Waals forces.

**Figure 7:**
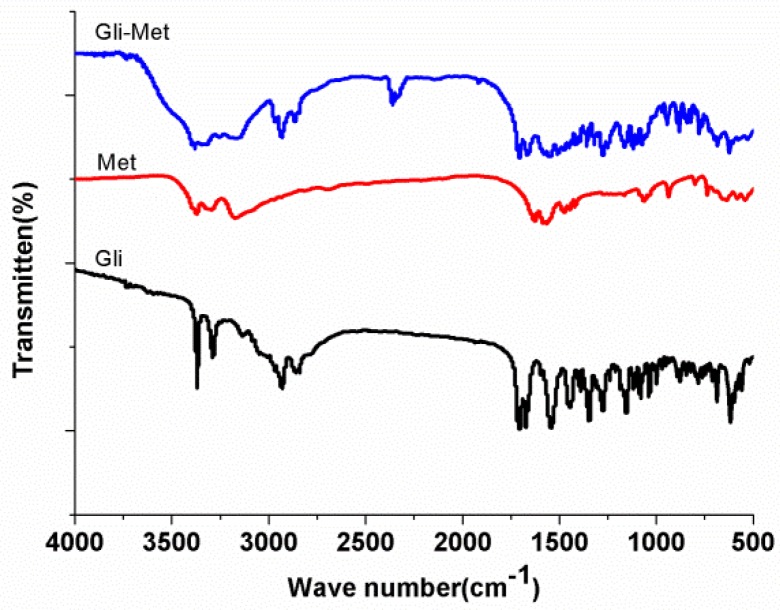
Infrared spectra of Gli-Met, Met and Gli.

Fourier-transform infrared (FT-IR) spectrum analysis was used an auxiliary method to detect differences between the Gli, Met, and Gli-Met samples at a molecular level. In the first instance, the Gli-Met IR spectrum should be a juxtaposition of the IR spectra of the parent compounds, with slight shifts for groups involved in strong hydrogen bonding. Additionally, the removal of the H atom of the amino group makes nitrogen become anionic. This can enhance the conjugative effect, which causes the absorption of the surrounding groups moving to a high frequency. This indeed was the case, as shown in Figure 7.

In the infrared spectra of the Gli-Met, N-H vibration(3371 cm^−1^) from Met moved to a high-frequency and showed a novel absorption peak at 3381 cm^−1^, which was influenced by the H atom of N between sulfonyl and carbamide from Gli moving to a biguanide bond from Met. The C=O vibration of sulfonyl and lactam (1708 cm^−1^ and 1673 cm^−1^) from Gli moved to a low-frequency and showed novel absorption peaks at 1704 cm^−1^ and 1665 cm^−1^, which were influenced by producing a slight electron pair effect due to the formation of N anions. These results manifest that Gli-Met formed hydrogen bonds and ionic bonds between Gli and Met. Our conclusion from the analysis according to the FT-IR spectrum was consistent with that from SCXRD.

### 2.2. The Characterization of Physicochemical Properties

**Figure 8:**
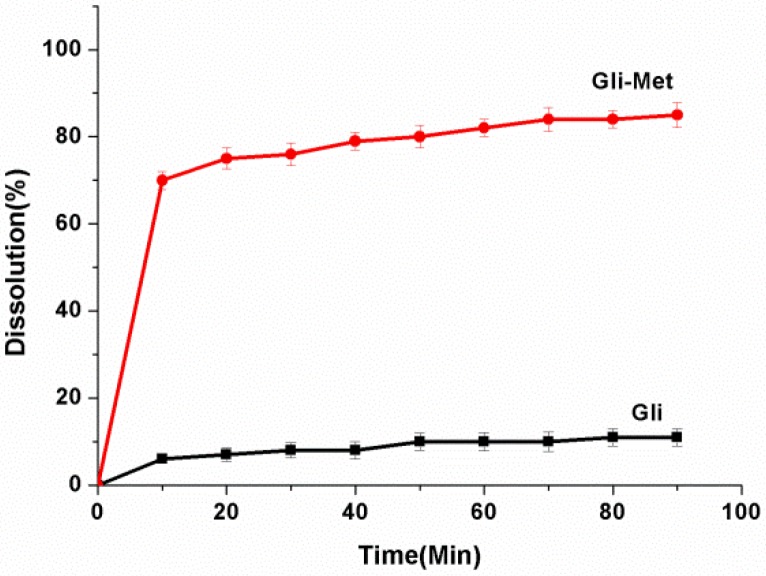
The dissolution rate of Gli-Met and Gli (pH = 1).

The first physicochemical evaluation of Gli-Met was through dissolution tests. Improving the poor dissolution of Gli is necessary because it is classified as a class II molecule, according to the BCS. Additionally, since Gli is a weak acid, the absorption of Gli in the gastrointestinal tract occurs predominantly in the stomach. Therefore, we conducted this experiment in an acidic medium (pH = 1). As shown in Figure 8 and Table S1, the dissolution rate of Gli-Met was significantly higher than that of the Gli raw material. Additionally, the released amounts were consistently higher for Gli-Met than for Gli alone during the 90 min period. Finally, the dissolution rate of Gli-Met reached equilibrium and peaked at its highest value around 80%.

Notably, the Met powder was directly soluble when it made contact with the acidic medium (pH = 1), and it was impossible to gain the data of the dissolution rate. Therefore, there was no group of Met in this test and we concluded that Met is an extremely soluble compound. In this context, the trend depicting the dissolution rates of these samples was as follows: Met > Gli-Met > Gli.

**Figure 9:**
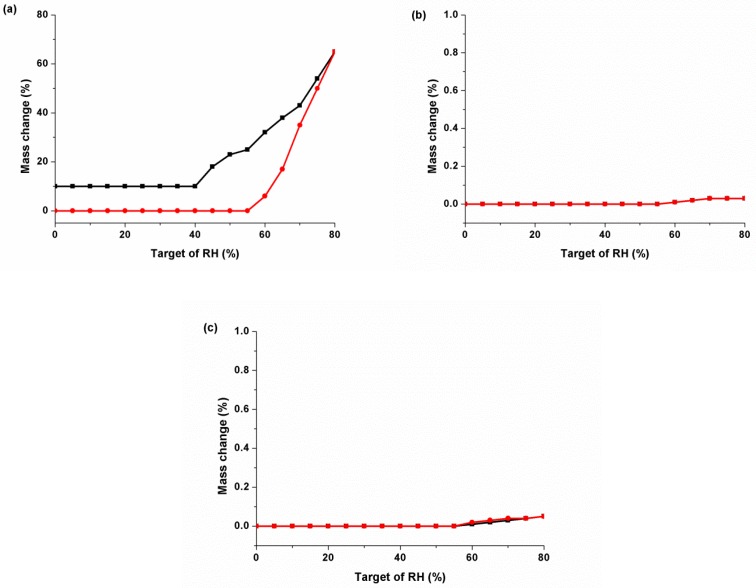
Dynamic vapor sorption charts of (**a**) Met, (**b**) Gli, and (**c**) Gli-Met. The red and black symbols represent absorption and desorption, respectively.

The other physicochemical evaluation of this multidrug crystal was sensitivity to water. Dynamic vapor sorption (DVS) analysis was performed to compare the hygroscopicity of Gli-Met with that of the two raw materials in Figure 9. Met powder has extremely hygroscopic properties, so this test was conducted at up to 80% relative humidity (RH). The patterns of Met started to absorb water at 55% RH and became deliquescence. On the other hand, the patterns of Gli showed nearly no change at an RH of up to 80%. This result was expected because Gli has poor solubility and hence low hygroscopicity. Interestingly, the patterns of Gli-Met exhibited a nonhygroscopic property. The water uptake at an RH of 80% was only 0.05%.

This could be explained by the model of Gli-Met structure, as shown in Figure 5. The Met and H_2_O molecules were located in the sandwich covered by the Gli molecules. Additionally, Gli, a less hydrophilic compound, could reduce the exposure of pure Met to water and the opportunities for the formation of hydrogen bonds Therefore, it is reasonable to conclude that the multidrug crystals showed a significant drop in hygroscopic property when compared with Met.

## 3. Materials and Methods

### 3.1. Materials

Gli was purchased from Adamas Reagent Company (Shanghai, China) and used as received. Met was prepared by adding metformin hydrochloride (0.65 g, 0.004 mol) and sodium hydroxide (0.1 g, 0.004 mol) into 70 mL of ethanol and the suspension was filtered after stirring at 25 °C for 12 h, followed by removing solvent with the rotary evaporator. The obtained free base of Met was freshly used in the next experiments. Other chemicals were purchased from Adamas Reagent Company (Shanghai, China), and used without any further purification.

### 3.2. Solvent Evaporation Method

The boiling ethanol (35 mL) containing Gli (2.00 g, 0.004 mol) was added to the same volume of ethanol containing Met (0.52 g, 0.004 mol) and vigorously stirred. The resulting solution was kept for three days at 25 °C, yielding colorless prism-shaped crystals that are suitable for single crystal X-ray diffraction. The obtained solids were filtered and dried under room condition for further characterization.

### 3.3. The Characterization of the Crystal Structure

#### 3.3.1. Powder X-ray Diffraction (PXRD)

PXRD data for the crystalline products were collected using a Bruker D8 Advance X-ray diffractometer (Bruker, Karlsruhe, Germany), operating in transmission geometry with Cu Kα radiation (λ = 1.5406 Å), 40 kV/100 mA. The samples were prepared on silicon single crystal sample holders with a 20 mm depth. Data for each sample were collected from 2θ = 5° to 50° at 25 °C with a step and scan speed of 5°/min.

#### 3.3.2. Thermogravimetric Analysis (TGA)

Thermogravimetric analysis was conducted in a NETZSCH STA 449 C (NETZSCH, Selb, Germany) using a nitrogen gas purge flow of 20 mL/min and a scan rate of 10 °C/min. The sample (10 mg) was placed into a hermetically sealed aluminum pan containing a pinhole. The sample cell was equilibrated at 25 °C and then heated to 500 °C. Indium metal was used as the calibration standard.

#### 3.3.3. Differential Scanning Calorimetry (DSC)

Every crystal drug has its intrinsic melting point, and the novel absorption peak can be detected with the emergence of a novel crystal by DSC. Differential scanning calorimetry analyses were carried out on a NETZSCH-TA4 STA Instruments 449C differential scanning calorimeter (NETZSCH, Selb, Germany). Each sample (5 mg) was placed into a hermetically sealed aluminum DSC pan containing a pinhole. The sample cell was equilibrated at 100 °C and then heated to 250 °C under a nitrogen purge at a rate of 10 °C/min. Indium metal was used as the calibration standard.

#### 3.3.4. Single Crystal X-Ray Data Collection and Structure Determinations

Single crystal X-ray diffraction (SCXRD) data were collected on a Bruker SMART CCD diffractometer (Bruker, Karlsruhe, Germany) using Cu-Kα radiation (λ = 1.54184 Å) with a graphite monochromator at 293 K. The integrated and scaled data were empirically corrected for absorption effects with spherical harmonics, implemented in the SCALE3 ABSPACK scaling algorithm. Using Olex2 [20], the structure was solved with the ShelXS [21] structure solution program using direct methods and refined with the ShelXL [22] refinement package using least squares minimization. All non-hydrogen atoms were refined with anisotropic displacement parameters. The hydrogen atoms were located from the differential Fourier map and refined with isotropic displacement parameters.

#### 3.3.5. Fourier-Transform Infrared (FT-IR)

Mortars and pestles were previously washed and placed in a dryer for 30 min. Then, each compound (1 mg) was ground into powder with dried KBr (50 mg) in a mortar. The mixture was pressed into a piece of slice and recorded on a Nicolet Spectrum FT-IR spectrometer (Nicolet iS10, Waltham, MA, USA) in the range of 4000–400 cm^−1^.

#### 3.3.6. Polarized Optical Microscopy (POM)

Several droplets of Met, Gli, and Gli-Met were deposited on a microscope slide for observation. Optical characterization of these samples were carried out at 22 °C by POM using an Olympus transmission microscope coupled with a Leica digital camera and Leica Application Suite Software.

### 3.4. The Characterization of Physicochemical Properties

#### 3.4.1. Dissolution Rate

The dissolution rate of pure Gli and Gli-Met in powder form were studied by using a U.S. pharmacopoeia tablet dissolution test apparatus (Hanson Research, America) at a paddle rotation speed of 100 rpm in 900 mL of 0.1 N HCl containing 0.25% (*w*/*v*) of sodium lauryl sulfate as a dissolution medium at 37.5 ± 0.5 °C. The powder equivalent to 100 mg of Gli was weighed and added into the dissolution medium. At specified times (every 10 min for 90 min), 10 mL samples were withdrawn by using a syringe with a nylon 0.45 µm filter (Titan, Shanghai, China). The content of Gli was measured at 273 nm for Gli and 224 nm for Gli-Met using a UV-Visible spectrophotometer (Shimadzu UV2600, Shimadzu, Japan). Fresh medium that was pre-warmed at 37 °C was added to maintain its constant volume. Dissolution rates were performed in triplicate.

#### 3.4.2. Dynamic Vapor Sorption (DVS)

The water sorption and desorption processes were measured on an Intrinsic DVS instrument (SMS Ltd., London, UK). Samples were mounted on a balance and studied over a humidity range from 0 to 80% relative humidity (RH) at 25 °C. Each humidity step was made if less than a 0.02% weight change occurred in 10 min, with a maximum hold time of 3 h.

## 4. Conclusions

In conclusion, we created a novel drug-drug pharmaceutical crystal, and the process of preparation of Gli-Met is simple and the reagents used in this experiment are cheap. This new multidrug exhibits dramatic changes in the physicochemical properties such as the hygroscopicity and solubility of the two raw materials. However, it is difficult to address the issue of solubility and hygroscopicity at the same time as these factors are contradictory in essence [23]. For example, if a certain technique can alter the solubility of the parent drugs, the hygroscopicity in the raw materials must be increased consequently, and vice versa.

To the best of our knowledge, this is the first report to show a drug–drug hydrous pharmaceutical crystal of Gli based on drug combinations. The work described above shows a promising method that can be used to overcome the poor physicochemical properties of the parent drugs by forming a sandwich crystal unit. Additionally the combination of these advantages makes it an alternative for use against type 2 diabetes as opposed to pure Met or Gli. Drug-drug pharmaceutical crystals based on drug combinations could be a major trend in the pharmaceutical field.

## Figures and Tables

**Table 1:** Crystal data and structure refinement for Gli-Met.

Empirical Formula	C_28_H_47_N_9_O_6_S
Formula weight	637.80
Temperature/K	293
Crystal system	monoclinic
Space group	P2_1_/c
*a*/Å	30.9830
*b*/Å	8.91714
*c*/Å	11.8813
α/°	90
β/°	98.8439
γ/°	90
Volume/Å^3^	3243.53
Z	4
ρcalcg/cm^3^	1.306
μ/mm^−1^	1.344
F(000)	1368.0
Crystal size/mm^3^	0.14 × 0.11 × 0.1
Radiation	CuKα (λ = 1.54184)
2θ range for data collection/°	10.332 to 134.158
Index ranges	−37 ≤ h ≤ 33, −10 ≤ k ≤ 10, −11 ≤ l ≤ 14
Reflections collected	12812
Independent reflections	5796 [*R*_int_ = 0.0317, *R*_sigma_ = 0.0421]
Data/restraints/parameters	5796/0/442
Goodness-of-fit on F^2^	1.025
Final *R* indexes [*I* > 2σ (*I*)]	R_1_ = 0.0470, wR_2_ = 0.1237
Final *R* indexes [all data]	R_1_ = 0.0611, wR_2_ = 0.1368
Largest diff. peak/hole/e Å^−3^	0.31/−0.34
CCDC no.	1954033

**Table 2:** Hydrogen bonds for Gli-Met.

D-H...A	d(D-H)/Å	d(H...A)/Å	d(D...A)/Å	D-H-A/°
N(2)-H(2A)...O(1)	0.83 (3)	2.18	2.841	137
N(3)-H(3A)...O(5)	0.88	2.00	2.720	138
O(6)-H(6C)...N(1) ^1^	0.88	2.12	2.988	166
N(5)-H(5A)...O(6)	0.84	2.14	2.988	178
N(5)-H(5B)...N(7) ^2^	0.91	2.19	3.083	167
N(6)-H(6A)...O(1) ^2^	0.86	2.26	3.086	161
N(6)-H(6B)...O(3) ^1^	0.93	1.89	2.758	154
N(8)-H(8C)...O(6) ^3^	0.84	2.24	2.982	148
N(8)-H(8D)...O(2) ^4^	0.87	2.07	2.863	152
O(6)-H(6D)...O(3) ^5^	0.84	2.19	3.022	173

Symmetry transformations used to generate equivalent atoms: ^1^ 1 − x, −y, 1 − z; ^2^ 1 − x, 1 − y, 1 − z; ^3^ +x, 1/2 − y, −1/2 + z; ^4^ 1 − x, 1/2 + y, 1/2 − z; ^5^ 1 − x, 1/2 + y, 3/2 − z.

## Supplementary Materials

The crystallographic information file (cif) of this study is deposited at the Cambridge Crystallography Data Center with deposit number 1954033. This data can be obtained free of charge from The Cambridge Crystallographic Data Centre via www.ccdc.cam.ac.uk/data_request/cif. Table S1. Dissociation rate and solubility of Gli-Met and Gli; Figure S1. The UV-Vis patterns of Gli-Met and Gli are also attached in the supplementary files.
