# Supplementary figures and images for: A Glimepiride-Metformin Multidrug Crystal: Synthesis, Crystal Structure Analysis, and Physicochemical Properties
Source: Molecules. 2019 Oct 21;24(20):3786. doi: 10.3390/molecules24203786 (PMC6832914; doi:10.3390/molecules24203786)

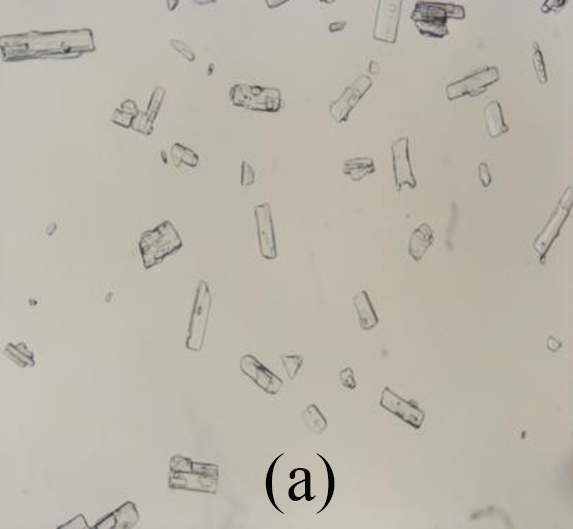

Supplement: Supplementary file 1 [file molecules-24-03786-s001.zip › figure3(a).tif]

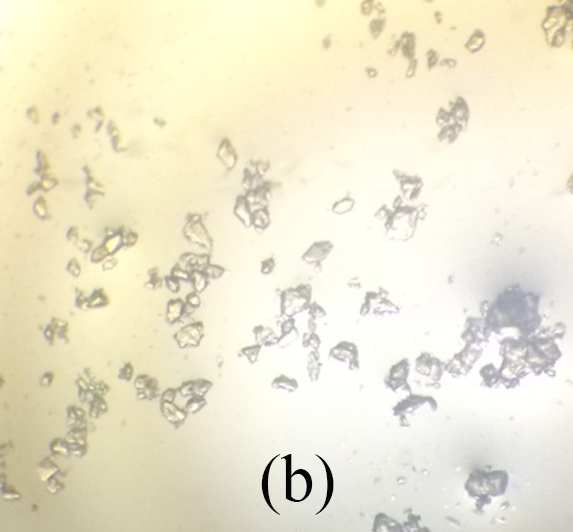

Supplement: Supplementary file 1 [file molecules-24-03786-s001.zip › figure3(b).tif]

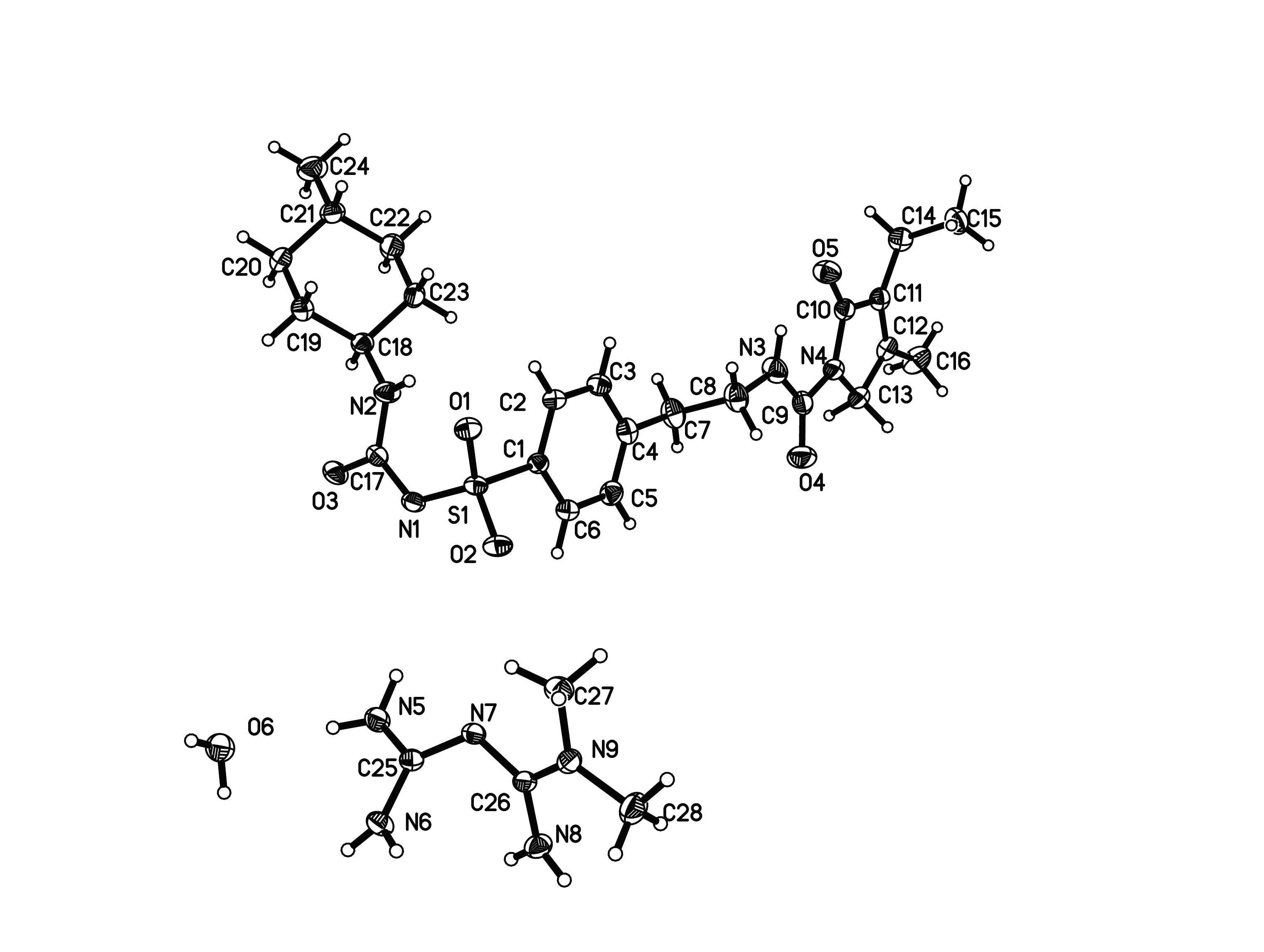

Supplement: Supplementary file 1 [file molecules-24-03786-s001.zip › figure4.tif]

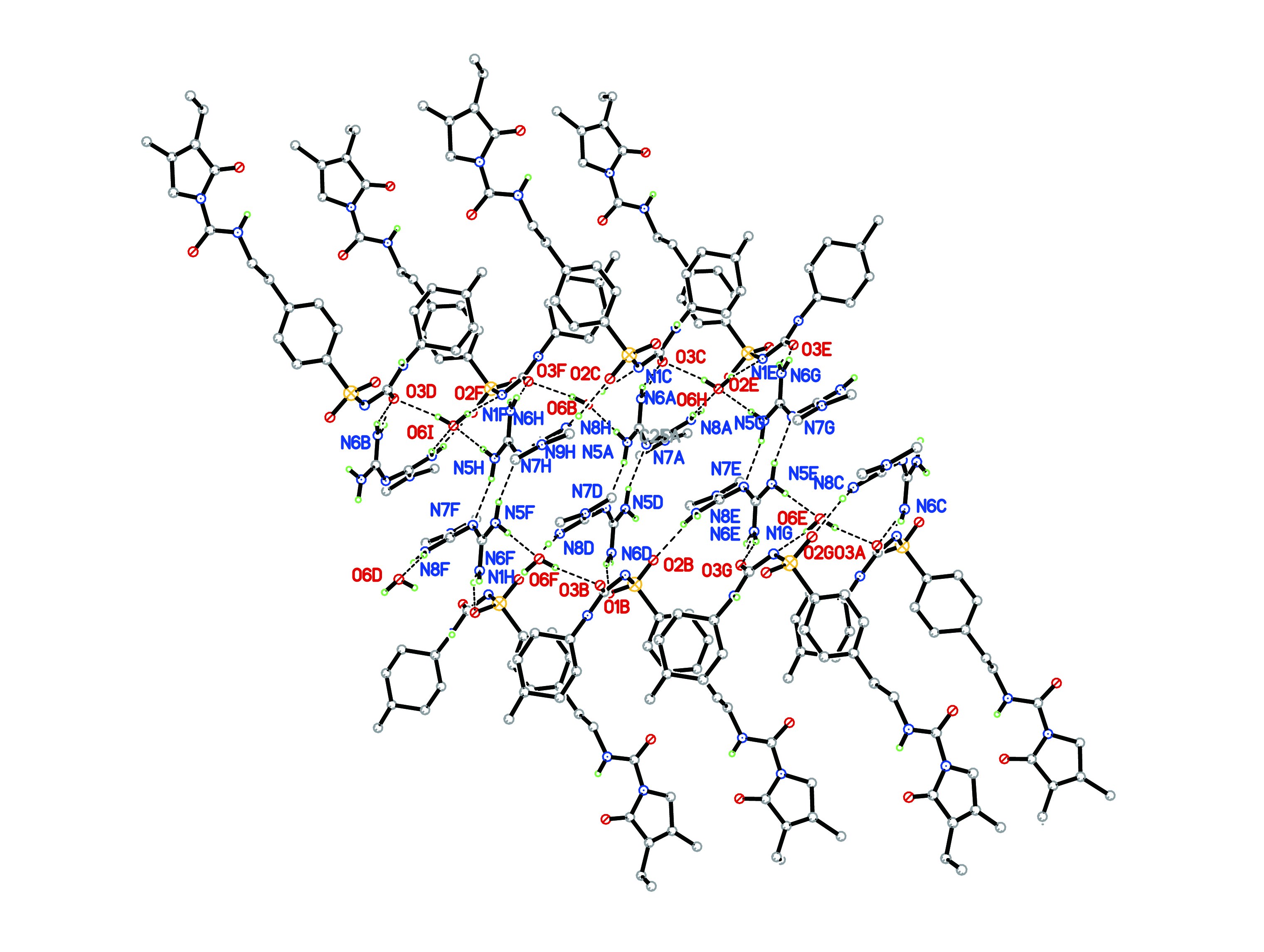

Supplement: Supplementary file 1 [file molecules-24-03786-s001.zip › figure5.tif]
